# Nitazoxanide and nonsteroidal anti-inflammatory drugs (NSAIDs): unveiling the connection in immediate hypersensitivity cross-reactions

**DOI:** 10.31744/einstein_journal/2025RC1534

**Published:** 2025-10-20

**Authors:** André Luiz Oliveira Feodrippe, Marcelo Vivolo Aun, Elaine Gagete, Jorge Kalil, Pedro Giavina-Bianchi

**Affiliations:** 1 Universidade de São Paulo Faculdade de Medicina Clinical Immunology and Allergy Division São Paulo SP Brazil Clinical Immunology and Allergy Division, Faculdade de Medicina, Universidade de São Paulo, São Paulo, SP, Brazil.; 2 Hospital Israelita Albert Einstein Faculdade Israelita de Ciências da Saúde Albert Einstein São Paulo SP Brazil Faculdade Israelita de Ciências da Saúde Albert Einstein, Hospital Israelita Albert Einstein, São Paulo, SP, Brazil.

**Keywords:** Nitazoxanide, Anaphylaxis, Hypersensitivity, Anti-inflammatory agents, nonsteroidal, Cross reactions, Salicylate, Salicylamide, Aspirin

## Abstract

Intestinal worm infections, referred to as helminthiases, represent a significant global health challenge, particularly in low- and middle-income countries. These infections affect millions of people and contribute to malnutrition, anemia, and impaired cognitive development, particularly in children. Nitazoxanide, first described in 1975, was initially developed as a veterinary anthelmintic. It has shown efficacy against various pathogens including *Giardia lamblia*, *Entamoeba histolytica*, and *Cryptosporidium parvum*. Recently, its use has expanded due to its observed in vitro activity against viruses, including coronaviruses. Nitazoxanide, a derivative of salicylamide, combines a nitrothiazole moiety with a salicylic acid moiety. Anaphylaxis, a severe immediate hypersensitivity reaction, may be induced by medications, with nonsteroidal anti-inflammatory drugs being among the most common causes. However, neither immediate hypersensitivity reactio, including anaphylaxis, to nitazoxanide nor cross-reactivity with nonsteroidal anti-inflammatory drugs has been previously reported. This study reports four cases of immediate hypersensitivity reactio to nitazoxanide in patients with non-selective immediate hypersensitivity reactio to nonsteroidal anti-inflammatory drugs. These findings highlight the need for vigilance in monitoring medication reactions and suggest the inclusion of nitazoxanide in the list of medications to be avoided in these patients.

## INTRODUCTION

Intestinal worm infections, referred to as helminthiases, represent a significant global health challenge, particularly in low- and middle-income countries. These infections affect millions of people and contribute to malnutrition, anemia, and impaired cognitive development, particularly in children. These infections are often exacerbated by inadequate sanitation, limited access to clean water, and insufficient healthcare infrastructure, conditions that are prevalent in many vulnerable communities. Effective treatment and preventive interventions, including periodic mass drug administration and improved hygiene practices, are essential for mitigating the burden of these infections. Addressing helminth infections not only enhances individual health outcomes but also supports broader public health goals, contributing to improved quality of life and economic stability in affected regions.^(1)^

Nitazoxanide, first described in 1975 by Rossignol et al., was initially developed as a veterinary anthelmintic with activity against intestinal nematodes, cestodes, and hepatic trematodes.^(2)^ Both nitazoxanide and its active metabolite (tizoxanide) exhibit a broad spectrum of pharmacological properties in humans, with literature demonstrating their efficacy against pathogens such as *Giardia lamblia*, *Entamoeba histolytica*, and *Cryptosporidium* parvum.^(3)^ Recently, the use of nitazoxanide has expanded, with observations of in vitro activity against different viruses, including coronaviruses,^(4–6)^ leading to an increase in its commercialization and utilization.

**Figure 1:**
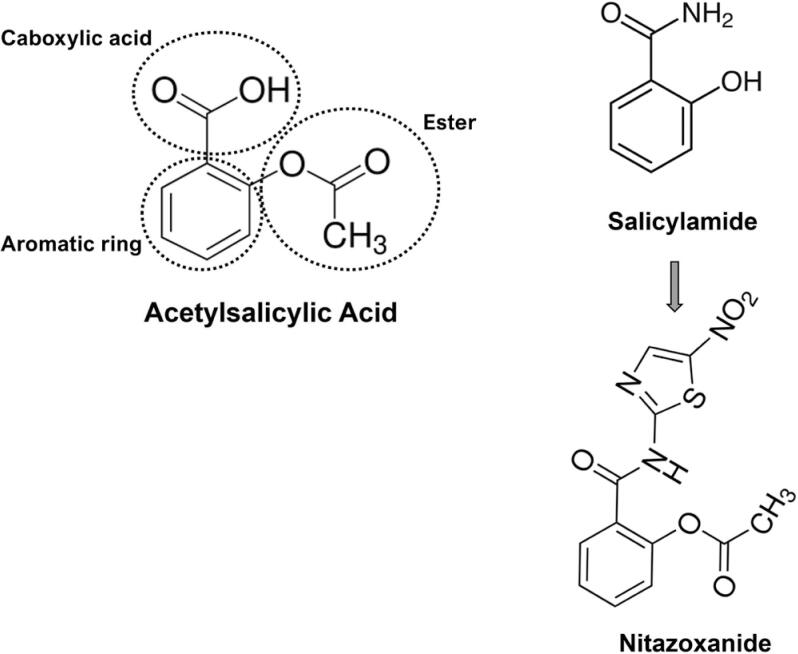
Chemical structure of nitazoxanide and its correlation with salicylamide and acetylsalicylic acid

Nitazoxanide is a derivative of salicylamide (2-hydroxybenzamide), a nonsteroidal anti-inflammatory drug belonging to the salicylate group. Members of the salicylate group include acetylsalicylic acid (aspirin), amoxiprin, benorylate, choline magnesium salicylate, diflunisal, ethenzamide, faislamine, methyl salicylate, magnesium salicylate, salsalate, salicyl salicylate, salicylamide, sodium salicylate, and sulfasalazine. Structurally, this molecule is composed of two groups, a nitrothiazole group and a salicylic acid group linked by an amide bond (Figure 1).^(7)^

Anaphylaxis is a medical emergency, representing a severe, immediate, potentially life-threatening systemic hypersensitivity reaction (iHSR). It results from mast cell and basophil degranulation and is classified into two main types: allergic, characterized by an IgE-mediated immune response, and non-allergic, involving direct activation of mast cells and basophils.^(8)^

Medications are major contributors to anaphylaxis, with nonsteroidal anti-inflammatory drugs (NSAIDs) increasingly recognized as a frequent cause of drug-induced anaphylaxis.^(9)^ Widely prescribed and globally used, NSAIDs are also associated with adverse hypersensitivity reactions that often require evaluation and management by allergists.^(10)^

To the best of our knowledge, no cases of iHSR, including anaphylaxis, related to nitazoxanide have been reported. Furthermore, there have been no reports of cross-reactions between this medication and NSAIDs. In this study, we report four cases of iHSR to nitazoxanide in patients exhibiting non-selective iHSR to NSAIDs.

## CASE REPORTS

Case 1: A 38-year-old male physician experienced urticaria, angioedema, and cough accompanied by sneezing, nasal obstruction, and ocular pruritus in January 2023. This iHSR occurred 30 minutes after the first oral dose of nitazoxanide (500 mg; Annitaä, Farmoquímica) taken for routine deworming without specific symptoms. The anaphylactic reaction was managed with an antihistamine (fexofenadine) and a corticosteroid (prednisone), resulting in complete resolution. The patient had a prior history of hypersensitivity reactions to multiple NSAIDs, manifesting as urticaria, angioedema, nasal obstruction, and cough. Comorbidities included rhinitis and asthma, and his only regular medication was vortioxetine.

Case 2: A 30-year-old female physician developed urticaria and angioedema approximately one hour after her second dose of nitazoxanide in July 2019. She was unable to recall the brand of the tablets. The iHSR was managed with antihistamines and corticosteroids. Nitazoxanide was prescribed for diarrhea. The patient also reported a prior hypersensitivity reaction to NSAIDs, characterized by urticaria and angioedema. Her comorbidities included chronic inducible urticaria (dermographic) and allergic rhinitis, and her only daily medication was atenolol.

Case 3: A 28-year-old female physician experienced two episodes of urticaria and angioedema 30 minutes after nitazoxanide intake, first in 2008 and again in April 2011, the latter occurring after ingestion of the first tablet of Annita™ (Farmoquímica). The medication was administered for routine deworming, without any specific symptoms. Both iHSRs were managed with antihistamines. She had a prior history of NSAID–induced reactions, presenting as urticaria, angioedema, sneezing, and ocular pruritus. Her only comorbidity was rhinitis, and she was not on any daily medications.

Case 4: A 58-year-old female reported recurrent episodes of facial angioedema and hives over the previous year, each episode lasting a few hours and resolving within 24 hours. Reactions were sometimes triggered by NSAIDs, while others occurred spontaneously. She had been treated with nitazoxanide for giardiasis without any reactions approximately 5 months earlier. Her eosinophil count was 750 cells/mm^3^. The patient experienced new abdominal pain and was administered another dose of nitazoxanide; 30 minutes after ingestion, she developed facial and tongue angioedema, generalized urticaria, and respiratory difficulty. She reported having taken nimesulide for low back pain approximately 3 hours before this episode. A provocation test with nitaxozanide suspension reproduced symptoms: 90 minutes after the full dose, she developed lip angioedema, urticarial papules on the face and neck, hoarseness, and mild dyspnea, without desaturation, bronchospasm, cough, or changes in blood pressure or heart rate. She was treated with intramuscular adrenaline (0.5ml, vastolateral thigh) and fexofenadine (180mg), with complete resolution after approximately 1 hour (Table 1).

**Table 1:** Case series of immediate hypersensitivity reactions to Nitaxozanide

Case	Gender/Age (y/o)	Characteristics of the iHSR to nitaxozanide	Treatment	iHSR to NSAIDs	Comorbidities	Concomitant medication
1	M / 38	Anaphylaxis	Antihistamine, Corticosteroid	Non-selective (anaphylaxis), N-ERD	Rhinitis, Asthma	Vortioxetine
2	F / 30	Urticaria, Angioedema	Antihistamine, Corticosteroid	Non-selective (urticaria, angioedema), N-ECD	Chronic inducible urticaria, Rhinitis	Atenolol
3	F / 28	Urticaria, Angioedema	Antihistamine	Non-selective induced (anaphylaxis)	Rhinitis	None
4	F / 58	Anaphylaxis	Antihistamine, Corticosteroid	Non-selective (angioedema) N-ECD	Chronic spontaneous urticaria. Diabetes, arterial hypertension, and dyslipidemia.	Nimesulide, 3 hours before the reaction. Losartan, metformin, and sinvastatin.

NSAIDs: nonsteroidal anti-inflammatory drugs; iHSR: immediate hypersensitivity reaction; N-ERD: nonsteroidal anti-inflammatory drug exacerbated respiratory disease; N-ECD: nonsteroidal anti-inflammatory drug exacerbated cutaneous disease.

The study was approved by CAPPesq, the ethical committee of the *Hospital das Clinicas da Faculdade de Medicina da Universidade de São Paulo* (CAAE: 38855420.0.0000.0068; # 4.381.117), and all patients provided informed consent for the publication of their data.

## DISCUSSION

Nitazoxanide, which was initially developed as a veterinary anthelmintic, has evolved into a versatile therapeutic agent with demonstrated efficacy against various pathogens, including parasites and viruses. The expansion of its applications, particularly during the global pandemic, underscores its evolving significance in medical practice. Structurally derived from salicylamide, nitazoxanide combines a nitrothiazole moiety with a salicylic acid moiety and exhibits a unique mechanism of action that directly affects extracellular parasites and inhibits PFOR, an enzyme critical for the anaerobic metabolism of specific microorganisms.^(7)^ While the broad pharmacological profile of nitazoxanide is well documented, there are few reports regarding iHSRs, including anaphylaxis, to nitazoxanide. In addition, no cross-reactions between nitazoxanides and NSAIDs have been reported. However, considering the significant role of NSAIDs as a common cause of drug-induced anaphylaxis, especially in Latin America, allergists frequently encounter cases of adverse reactions with this medication group.^(9)^ This study contibutes valuable information by presenting four cases of immediate hypersensitivity reactions to nitazoxanide in patients who had already exhibited non-selective immediate hypersensitivity reactions to NSAIDs.

These findings underscore the need for continual vigilance in monitoring adverse reactions to medications and advocate the inclusion of nitazoxanide in the list of medications to be avoided in patients with non-selective iHSR to NSAIDs. With the evolving landscape of drug reactions, ongoing research and heightened awareness among healthcare professionals is imperative to ensure optimal patient care and safety.

### Declaration of generative AI and AI-assisted technologies in the writing process

During the preparation of this work the author(s) used ChatGPT to review the writing of the manuscript. After using this tool/service, the authors reviewed and edited the content as needed and takes full responsibility for the content of the publication.

## References

[1] Wolf J, Johnston RB, Ambelu A, Arnold BF, Bain R, Brauer M (2023). Burden of disease attributable to unsafe drinking water, sanitation, and hygiene in domestic settings: a global analysis for selected adverse health outcomes. Lancet.

[2] Rossignol JF, Cavier R (1975). New derivative of 2-benzamido-5-nitrothiazoles. Chem Abstr.

[3] Abaza H, El-Zayadi AR, Kabil SM, Rizk H (1998). Nitazoxanide in the treatment of patients with intestinal protozoan and helminthic infections: a report on 546 patients in Egypt. Curr Ther Res.

[4] Rossignol JF (2014). Nitazoxanide: a first-in-class broad-spectrum antiviral agent. Antiviral Res.

[5] Rossignol JF (2016). Nitazoxanide, a new drug candidate for the treatment of Middle East respiratory syndrome coronavirus. J Infect Public Health.

[6] Xu J, Xue Y, Bolinger AA, Li J, Zhou M, Chen H (2023). Therapeutic potential of salicylamide derivatives for combating viral infections. Med Res Rev.

[7] Broekhuysen J, Stockis A, Lins RL, De Graeve J, Rossignol JF (2000). Nitazoxanide: pharmacokinetics and metabolism in man. Int J Clin Pharmacol Ther.

[8] Giavina-Bianchi P, Aun MV, Kalil J (2018). Drug-induced anaphylaxis: is it an epidemic?. Curr Opin Allergy Clin Immunol.

[9] Aun MV, Blanca M, Garro LS, Ribeiro MR, Kalil J, Motta AA (2014). Nonsteroidal anti-inflammatory drugs are major causes of drug-induced anaphylaxis. J Allergy Clin Immunol Pract.

[10] Giavina-Bianchi P, Aun MV, Jares EJ, Kalil J (2016). Angioedema associated with nonsteroidal anti-inflammatory drugs. Curr Opin Allergy Clin Immunol.

